# Endometrial Carcinoma and Associated Secondary Neoplasia: The Role of Clinical Features, Pathology, and Comorbidities in a University-Affiliated Clinical Center from Western Romania

**DOI:** 10.3390/medicina61101748

**Published:** 2025-09-25

**Authors:** Ioana Hurmuz, Robert Barna, Bianca Natarâș, Iuliana-Anamaria Trăilă, Denisa Anderco, Sorin Dema, Aura Jurescu, Dorela-Codruța Lăzureanu, Sorina Tăban, Alis Dema

**Affiliations:** 1 Department of Microscopic Morphology-Anatomic Pathology, ANAPATMOL Research Center, “Victor Babes” University of Medicine and Pharmacy, 300041 Timisoara, Romania; ioana.hurmuz@umft.ro (I.H.); robert.barna@umft.ro (R.B.); bianca.nataras@umft.ro (B.N.); jurescu.aura@umft.ro (A.J.); lazureanu.dorela@umft.ro (D.-C.L.); taban.sorina@umft.ro (S.T.); dema.alis@umft.ro (A.D.); 2 Department of Pathology, “Pius Brînzeu” County Clinical Emergency Hospital, 300723 Timisoara, Romania; denisa_dobre@umft.ro; 3 Department of Radiotherapy, Emergency City Clinical Hospital Timisoara, 300079 Timisoara, Romania; sorin.dema@umft.ro; 4 Department of Oncology, “Victor Babes” University of Medicine and Pharmacy, 300041 Timisoara, Romania

**Keywords:** endometrial carcinoma, dual primary tumors, comorbidities in uterine cancer, breast and ovarian cancer association

## Abstract

*Background and Objectives*: Multiple primary malignancies involving endometrial carcinoma (EC) present complex diagnostic and management challenges. This study aimed to identify clinical, pathological, and demographic patterns among patients with EC and a second primary tumor and assess the role of comorbidities in tumor behavior. *Materials and Methods*: We retrospectively analyzed 35 women diagnosed with EC and a second malignancy between 2017 and 2024. We evaluated clinical variables, tumor characteristics, and comorbidities. Statistical analysis included chi-square tests, Mann–Whitney U tests, Kruskal–Wallis tests, Spearman correlations, and logistic regression. Multiple testing correction was applied using the Benjamini–Hochberg method. *Results*: Endometrioid EC was the most prevalent subtype (80%), most frequently associated with breast (28.5%) and colorectal cancers (11.4%). Obesity (45.7%), hypertension (62.9%), and diabetes (22.9%) were common. While univariate analysis suggested associations between comorbidities and tumor features (e.g., obesity and tumor type, ρ = 0.30, *p* = 0.08), no correlation remained significant after adjustment. Logistic regression identified age (OR = 0.88, CI: 0.79–0.98, *p* = 0.022) and obesity (OR = 0.11, CI: 0.01–0.83, *p* = 0.033) as independent predictors of non-endometrioid histology. *Conclusions*: These findings suggest that age and obesity may influence histological differentiation in EC with multiple primaries. This study suggest that age and obesity may play a role in the histological differentiation of EC in patients with multiple primary tumors. The small cohort size (*n* = 35) limits the statistical power and generalizability of the results; therefore, they should be regarded as exploratory and hypothesis-generating, warranting validation in larger prospective studies.

## 1. Introduction

The phenomenon of multiple primary malignancies in a single individual was recognized at the end of the 19th century. Billroth reported the first case of double primary cancers in 1869 [1,2]. Endometrial carcinoma (EC) mainly affects women in the postmenopausal period and is commonly associated with ovarian, colorectal, and breast cancers, as highlighted in various studies [3,4,5]. The occurrence of synchronous and metachronous tumors is driven by genetic factors, metabolic and hormonal influences, and the effects of treatments administered for the initial cancer [1,6].

Multiple primary malignant tumors refer to the development of distinct malignancies originating from different tissues that arise independently [7]. They are categorized as either synchronous or metachronous. Synchronous cancers emerge within six months of the first primary tumor, whereas metachronous cancers develop beyond this six-month timeframe [8].

Uterine carcinomas, especially the endometrioid type, are the most frequently diagnosed gynecological malignancy in high-income countries [9]. It is relatively common for patients with EC to develop a second primary malignancy or for individuals with other cancers to develop EC subsequently. This can be attributed to factors such as genetic disorders, longer life expectancy, and shared risk factors for various cancers, both environmental and biological. Conditions like hypercholesterolemia, diabetes, obesity, and a high level of estrogen increase the likelihood of tumor development. Hormonal therapy is another significant risk factor, while immunosuppression—whether caused by medical treatments or extensive surgical procedures—also plays a critical role. Suppressed immune function may heighten the risk of a secondary malignancy. Immunosuppressed individuals, in particular, show a higher incidence of squamous cell skin cancers and non-Hodgkin lymphomas [6,10].

Obesity is one of the most significant risk factors for endometrial cancer, with studies indicating that approximately 57% of cases can be directly attributed to it [11]. The increase in the prevalence of obesity is expected to contribute to an increase in endometrial cancer incidence. [12]. The relationship between obesity and endometrial cancer is primarily mediated through elevated estrogen levels, as adipose tissue can convert androgens into estrogens, leading to prolonged exposure of the endometrium to estrogen without the counterbalancing effects of progesterone [12,13,14].

Moreover, metabolic syndrome components, type 2 diabetes (T2D), and hypertension have been shown to increase the risk of endometrial cancer independently. Diabetes, particularly T2D, has been associated with an increased risk of endometrial cancer, possibly due to the hyperinsulinemic state that promotes cell proliferation and inhibits apoptosis in the endometrial tissue [15]. The mechanisms underlying the association between EC and these diseases may involve inflammatory pathways and insulin signaling, which are often dysregulated in individuals with metabolic syndrome [13,15,16].

Genetic predispositions also play a critical role in the risk of developing endometrial cancer. Lynch syndrome, caused by germline mutations in DNA mismatch repair genes, significantly increases the risk of endometrial cancer, accounting for 2–6% of all cases [17]. Furthermore, polymorphisms in genes related to estrogen metabolism and signaling, such as CYP19A1, have been implicated in endometrial cancer susceptibility, suggesting that individual genetic makeup can influence cancer risk in the context of hormonal exposure [18].

The impact of lifestyle factors on the risk of endometrial cancer cannot be overlooked. Regular physical activity has been associated with a reduced risk of endometrial cancer, potentially due to its effects on body weight and insulin sensitivity [19,20]. Conversely, sedentary behavior and poor dietary habits, characterized by high glycemic load and low nutrient density, have been linked to an increased risk of endometrial cancer [19,20].

The therapy of multiple malignancies necessitates a multidisciplinary approach in which oncologists, gynecologists, and genetic counselors collaborate to manage these patients comprehensively.

This study aims to answer the clinical question: Are there identifiable patient characteristics or comorbidities that predict the occurrence of dual primary malignancies involving endometrial carcinoma? We hypothesize that specific clinical or metabolic comorbidities (e.g., obesity, diabetes) are associated with a higher risk of synchronous or metachronous tumor development in women with EC. By analyzing demographic, pathological, and clinical data, we seek to identify patterns that may inform future screening and management strategies in this patient population.

## 2. Materials and Methods

### 2.1. Study Design and Participants

This retrospective, observational, and descriptive study was conducted at Victor Babes University of Medicine and Pharmacy, Timisoara, Romania, over seven years, from January 2017 to August 2024, with approval from the research ethics committee (No. 11, 8 January 2025) and the local ethics committee (No. 533, 11 February 2025).

Patients were identified by systematically reviewing all medical records and observation charts of women diagnosed with endometrial carcinoma between January 2017 and August 2024 at the Emergency County Clinical Hospital ‘Pius Brînzeu’ in Timisoara. Due to the rarity of dual primary malignancies involving endometrial carcinoma, especially within a single institutional setting, this study includes all eligible cases identified over 8 years (2017–2024). 35 cases were selected based on the inclusion criteria for the study:

- Adult women (≥18 years);
- Histopathologically confirmed endometrial carcinoma (as first or second primary tumor);
- Presence of a second histopathologically distinct primary malignancy at a non-uterine site;
- Availability of complete medical records, including tumor staging, histological details, and documentation of associated comorbidities.

Exclusion criteria were:

- Cases with suspected metastasis rather than an actual second primary tumor;
- Incomplete clinical or histopathological records;
- Cases with synchronous endometrial and ovarian tumors that did not meet Blaustein’s histological criteria for synchronous primaries.

Cases of synchronous endometrial and ovarian carcinomas were evaluated according to Blaustein’s pathology criteria [21]. These included:

- Low-grade endometrioid histology in both tumors.
- Absence of deep myometrial or lymphovascular invasion in the uterine tumor.
- Ovarian tumor features include expansive invasion, unilateral localization, and association with endometriosis.

Features suggesting metastasis (and thus exclusion) were:

- Bilateral ovarian involvement, small size, multinodular morphology, destructive stromal invasion, and lack of endometriosis or Müllerian borderline components.

No personal data was collected, ensuring participant confidentiality and adherence to ethical guidelines.

The compiled and analyzed dataset includes comprehensive information such as patients’ age, residential background, histopathological classification of the uterine tumor, and its FIGO (Fédération Internationale de Gynécologie et d’Obstétrique) grade and stage. It also details the type and stage of the second primary malignancy, the age at diagnosis for both tumors, and their temporal relationship (i.e., whether the tumors occurred synchronously or sequentially). The dataset also documents associated comorbidities, including obesity, T2D, hypertension, hypercholesterolemia, and hypertriglyceridemia, among others.

We used ChatGPT-5 (the latest version, GPT-5, by OpenAI, released on 7 August 2025) to improve and correct the text in English.

### 2.2. Statistical Analyses

Statistical analyses were conducted using Python 3.8 (Google Colaboratory environment). Mann–Whitney U, Kruskal–Wallis, and chi-square tests were applied appropriately for univariate analysis. *p*-values were adjusted using the Benjamini–Hochberg false discovery rate (FDR) method to reduce the risk of false-positive findings due to multiple comparisons (adjusted *p* > 0.1 in all cases). *p*-value is considered significant <0.05.

Additionally, a multivariate binary logistic regression model was used to explore the association between tumor histological type (endometrioid vs. non-endometrioid) and potential predictors, including age at diagnosis, obesity, and T2D. The dependent variable was coded 1 for endometrioid histology and 0 for other histologic types.

## 3. Results

We collected 36 cases for the cohort examined in this study, diagnosed with double primary tumors, one of them uterine, in the last 7 years from Emergency County Hospital “Pius Brânzeu”, Timișoara, Romania. We examined the data through a multifaceted approach with meticulous care.

### 3.1. Descriptive Statistics

Table 1 presents the demographic and clinical distribution of the cohort. Most patients had endometrioid tumors (80%), while non-endometrioid types were relatively rare. Grade 2 tumors were the most prevalent (60%), and early-stage FIGO stages IA and IB were the most frequently encountered. Although a chi-square test initially suggested a borderline significant variation across tumor types (*p* = 0.0551), this association lost statistical significance after adjusting for multiple comparisons (adjusted *p* = 0.2057).

In terms of associated malignancies, the most frequent secondary tumor was invasive ductal breast carcinoma (28.57%), followed by rectosigmoid adenocarcinoma and endometrioid ovarian tumors. While the unadjusted analysis showed a significant association (*p* = 0.0201), this also became non-significant after FDR correction (adjusted *p* = 0.1752). These findings underscore the importance of correcting for multiple testing in studies involving small cohorts and multiple categorical comparisons.

The sequence of tumor development showed that eight patients (22.85%) had uterine tumors as their first malignancy, while 14 (40.00%) had another organ site as their first malignancy. Synchronous tumors were observed in 12 patients (34.28%), and one case of triple malignancy was identified (2.85%).

**Figure 1:**
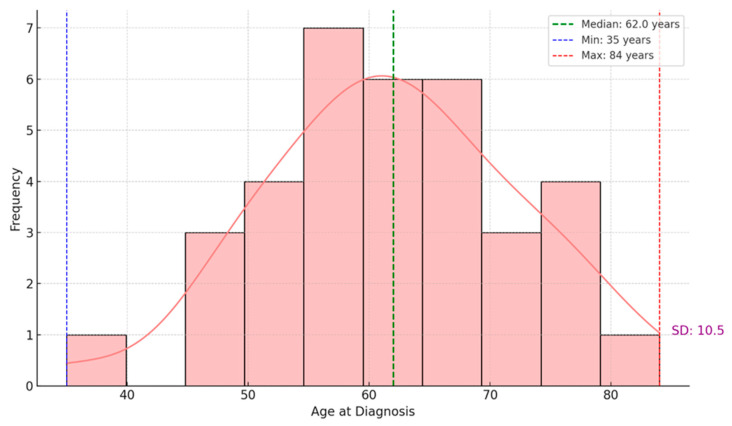
Age distribution of patients at diagnosis.

Regarding the age of patients at the time of diagnosis, we observed a median age of 62, indicating that half of the patients were diagnosed before this age. In contrast, the other half were diagnosed afterward. The youngest patient was 35, and the oldest was 84. The standard deviation of 10.5 years suggests moderate variability in the ages of the patients in this dataset. The age distribution follows an approximately regular pattern, with a peak in the 60- to 70-year age range. The density curve (KDE) overlaid on the frequency histogram highlights this trend, indicating that most patients were diagnosed between the ages of 50 and 70 (Figure 1).

Age variability differed across the histological types of uterine tumors. Endometrioid tumors, which were the most frequent, exhibited the widest age distribution, encompassing patients from approximately 35 to over 80 years. In contrast, serous and mesonephric-like tumors tended to occur more frequently at older ages, with narrower age distributions and fewer cases.

### 3.2. Analysis of Dual Primary Tumors Association

The distribution of primary malignant tumors associated with uterine malignancies based on their time of occurrence shows that in the group of patients where the uterine tumor was diagnosed first (8 cases), the most frequently identified second primary malignancy was infiltrative ductal breast carcinoma, followed by serous ovarian adenocarcinoma. For the group in which the first diagnosed primary tumor originated in a non-uterine location, the highest prevalence was observed for invasive ductal breast carcinoma and colorectal adenocarcinoma.

This category of synchronous tumors demonstrated a wide variety of tumor types, indicating a predisposition for the simultaneous diagnosis of multiple malignancies, including small-cell neuroendocrine lung carcinoma and malignant melanoma.

A single case of triple malignancy was recorded, comprising endometrioid endometrial carcinoma, endometrioid ovarian adenocarcinoma, and invasive ductal breast carcinoma. The associated tumors, in this case, were clinically significant, underscoring the rarity and complexity of such diagnoses.

Subsequently, two non-parametric tests were conducted: a Mann–Whitney U test to compare the ages at diagnosis between groups with different primary tumor sequences, and a Kruskal–Wallis test to assess the association between the time interval of tumor occurrence and tumor-related variables (histologic type, grade, and stage). The Mann–Whitney U test found no significant age differences between the groups (U = 51.0, *p* = 0.76). Conversely, the Kruskal–Wallis test showed a statistically significant link between the time interval separating the two malignancies and tumor characteristics (H = 6.91, *p* = 0.0002; FDR-adjusted *p* = 0.0036). These findings suggest that the time spacing between tumor diagnoses may be associated with differences in histologic subtype or disease stage (Table 2).

### 3.3. Correlation Between Comorbidities and Uterine Tumor Features

Among the 35 patients with dual primary tumors, obesity was the most prevalent comorbidity (45.7%), followed by hypertension (62.9%), hypercholesterolemia (37.1%), and diabetes mellitus (22.9%). Less common conditions included chronic kidney disease (5.7%), ischemic heart disease (5.7%), Parkinson’s disease, and stroke (2.9% each).

**Figure 2:**
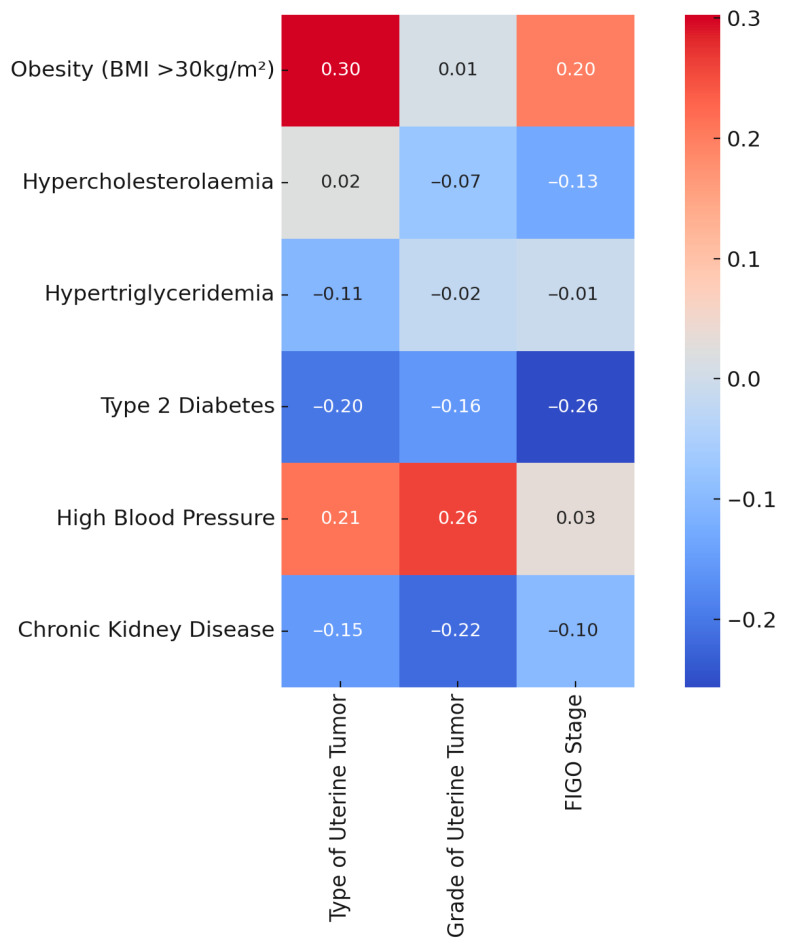
Spearman correlation heatmap: comorbidities vs. uterine tumor features.

Spearman’s correlation heatmap illustrating associations between comorbidities (e.g., obesity, hypertension, diabetes) and tumor characteristics (histologic type, grade, FIGO stage). Correlation coefficients are shown, though none reached statistical significance after FDR correction. Due to the small sample size, these findings are considered an exploratory analysis. Figure 2 shows a moderate, but statistically nonsignificant, association between obesity and tumor type (ρ = 0.30, *p* = 0.08; adjusted *p* = 0.2057). All other correlations with tumor grade or FIGO stage, including those involving diabetes, hypertension, hypercholesterolemia, and chronic kidney disease, were weak and not statistically significant after adjustment for multiple comparisons.

### 3.4. Analysis of Synchronous Tumor Cases

Thirteen patients were identified with synchronous primary tumors, representing a clinically relevant subset of the study cohort. Their demographic and clinical characteristics are summarized in Table 3. The majority of patients (76.9%) resided in rural areas. Endometrioid tumors were the most common histological type, found in 11 cases (84.6%), while serous and mesonephric-like tumors were each observed in one case. Although the unadjusted chi-square test showed a statistically significant variation in tumor type distribution (*p* = 0.0292), this association lost significance after correction for multiple comparisons (adjusted *p* = 0.1752).

Grade 2 tumors were the most frequent (61.5%), and the most common associated malignancies in this subgroup were ovarian tumors of varying histology, including endometrioid, serous, seromucinous, and mesonephric-like types.

Spearman correlation analysis revealed that obesity had the strongest positive correlation with the type of synchronous uterine tumor (ρ = 0.53), though this association did not reach statistical significance (*p* = 0.078). Other comorbidities, including hypercholesterolemia, hypertension, and diabetes, demonstrated weak or inconsistent correlations with tumor characteristics, none of which were statistically significant. These findings suggest possible trends but emphasize the need for larger cohorts to validate these associations.

### 3.5. Multivariate Logistic Regression Analysis

A multivariate logistic regression analysis was performed to identify independent predictors of endometrioid uterine tumor histology, with age at diagnosis, obesity, and T2D included as covariates (Table 4).

The analysis showed that increasing age was significantly associated with a lower likelihood of endometrioid histology (OR = 0.88, 95% CI: 0.79–0.98, *p* = 0.022), indicating that with each additional year of age, the odds of having an endometrioid tumor decreased. Similarly, obesity was also significantly associated with reduced odds of endometrioid tumor type (OR = 0.11, 95% CI: 0.01–0.83, *p* = 0.033).

T2D was not significantly associated with tumor histology (OR = 0.90, 95% CI: 0.12–6.68, *p* = 0.915).

These results suggest that non-endometrioid tumor types may be more common in older and obese patients, independent of diabetic status.

## 4. Discussion

In this study, the demographic, pathological, and clinical features of a selected group of patients diagnosed with uterine carcinoma and a second primary tumor were explored. We aimed to analyze the possible correlations between comorbidities and the dual malignancy profile of the patients and determine the impact of age, associated neoplasms, and tumor types on EC. The occurrence of multiple primary malignancies involving endometrial carcinoma is rare, which inherently limits the cohort size. This issue may not lend itself to broad generalizations. Still, it provides valuable insight into possible clinical patterns and associations, serving as a hypothesis-generating study for future multicenter or population-based research.

Obesity, T2D, and hypertension are well-established risk factors for endometrial carcinoma [22]. These conditions promote chronic low-grade inflammation, insulin resistance, and increased peripheral estrogen production from adipose tissue, all of which contribute to estrogen-driven carcinogenesis. Additionally, hyperinsulinemia and altered adipokine signaling may enhance cellular proliferation and reduce apoptosis. These shared metabolic pathways may also facilitate the development of secondary malignancies, particularly in hormone-responsive tissues such as the breast and ovary [23,24].

The analyzed cohort comprised 35 patients with a confirmed diagnosis of uterine carcinoma and a second primary neoplasm, synchronous or metachronous. The results revealed a predominance of endometrioid tumors (80%), and in most cases, the endometrial tumors were the first diagnosed.

Breast and colorectal carcinomas were the most common associated malignancies. Comorbidities such as obesity, diabetes, and hypertension were frequently observed. While some associations appeared suggestive in univariate testing, they did not retain significance after multiple testing correction. However, multivariate logistic regression identified age and obesity as independent predictors of histological subtype.

In this cohort, endometrioid carcinoma was the most common histological subtype. This aligns with large-scale epidemiological studies, which consistently identify endometrioid adenocarcinoma as the predominant histologic variant of endometrial cancer, particularly in early stages and among patients with metabolic risk [17,19,25,26,27]. The high prevalence of this subtype reinforces its strong association with estrogen-driven pathogenesis and supports previous reports from both Western and Eastern European populations [3,28].

The most frequently associated secondary malignancies were breast and colorectal cancers, a pattern that reflects known hormonal and genetic overlaps. Prior studies have documented a bidirectional risk between endometrial and breast cancer, partly mediated by shared risk factors such as obesity and tamoxifen exposure [29,30,31,32,33]. Additionally, the co-occurrence of endometrial and colorectal cancers raises the possibility of underlying Lynch syndrome, even in patients without confirmed genetic testing [34]. Findings from this analysis support these associations and emphasize the need for thorough family history assessments and, when appropriate, germline mutation screening.

Although rare, synchronous endometrial and ovarian carcinomas were observed in our study, often with endometrioid histology in both sites. This pattern has been previously reported in up to 10% of endometrial cancer cases, with synchronous ovarian involvement usually exhibiting similar differentiation and molecular signatures [32,35,36]. Blaustein’s histologic criteria remain a validated method for distinguishing actual synchronous primaries from metastatic spread. This study’s data reflects the diagnostic challenge in such cases and highlights the importance of histopathological review and multidisciplinary evaluation.

Obesity, hypertension, and T2D were prevalent in our cohort, consistent with their established role as significant risk factors for endometrial cancer. The literature consistently shows that metabolic syndrome components contribute to the development of estrogen-dependent tumors by promoting chronic inflammation, insulin resistance, and increased peripheral estrogen synthesis [1,12,13,26,37,38,39,40]. Although the univariate analyses suggested potential correlations, these did not remain statistically significant after FDR correction. Still, the high prevalence underscores the need for comprehensive metabolic evaluation in patients with EC, especially when a second malignancy is present.

Initial associations between specific comorbidities and tumor characteristics appeared suggestive in univariate analysis. For example, obesity showed a moderate correlation with histologic subtype (ρ = 0.30, *p* = 0.08), and the presence of multiple comorbidities appeared more frequent in non-endometrioid cases. However, after adjusting for multiple comparisons using the Benjamini–Hochberg false discovery rate (FDR), none of these associations remained statistically significant. This highlights a key limitation in exploratory research using small samples and multiple hypothesis testing: the risk of false positives. By applying FDR correction, our analysis reduced this bias and increased the reliability of reported associations.

Unlike univariate testing, the multivariate logistic regression model enabled us to evaluate the independent effects of multiple predictors on tumor histology. Notably, both increasing age and obesity were significantly associated with a lower likelihood of endometrioid histology. These associations remained robust even after adjusting for diabetes status, suggesting that these variables may directly influence tumor phenotype, not merely through metabolic intermediates. The logistic regression model identified age and obesity as independent predictors of histological subtype. Specifically, increasing age was associated with a 12% reduction in the likelihood of endometrioid histology per year (OR = 0.88), which is consistent with reports linking older age to more aggressive, non-endometrioid subtypes. By contrast, the finding that obesity was associated with a markedly lower probability of endometrioid tumors (OR = 0.11) is unexpected and contrary to the existing literature, which generally associates obesity with estrogen-driven type I endometrioid carcinomas. We interpret this as a potential artifact related to the small sample size and the retrospective single-center design, possibly compounded by unmeasured confounding variables or unique characteristics of the regional population. Therefore, this result should not be regarded as definitive but rather as a hypothesis-generating observation that warrants validation in larger, prospective cohorts. Such findings are consistent with other studies that have reported age-related shifts in tumor biology, including a greater proportion of aggressive non-endometrioid subtypes in older patients [41,42]. The inverse association with obesity is somewhat unexpected and may reflect complex interactions between body composition, age, and tumor evolution.

Although the logistic regression model produced statistically significant predictors, its interpretation must consider the modest sample size and the retrospective design. Wide confidence intervals, particularly for the diabetes variable (OR = 0.90, 95% CI: 0.12–6.68), indicate uncertainty in the effect estimate. This reflects limited statistical power and highlights the need for larger, prospective cohorts to validate these findings. Nonetheless, the consistent direction and strength of associations for age and obesity add credibility to the model and support its use as a preliminary tool for hypothesis generation.

The observed association between the interval separating tumor diagnoses and tumor characteristics may reflect underlying biological differences in tumor aggressiveness or host susceptibility. Shorter diagnostic intervals could indicate a predisposition to synchronous or rapidly emerging metachronous tumors, potentially driven by hereditary cancer syndromes (e.g., Lynch syndrome) or by shared metabolic and hormonal risk factors that accelerate tumorigenesis. Conversely, longer intervals may be more compatible with indolent tumor behavior and later-stage carcinogenic events.

From a clinical perspective, our findings highlight the importance of customizing follow-up and management approaches for patients with dual primary malignancies involving EC. The discovery of age and obesity as independent predictors of non-endometrioid histology indicates that these patients may benefit from more intensive monitoring, including closer imaging follow-up and earlier screening for secondary cancers like breast and colorectal cancer. Additionally, the common presence of metabolic comorbidities emphasizes the need for multidisciplinary care that combines oncologic, metabolic, and lifestyle interventions, aiming to improve both cancer-related outcomes and overall survival. Integrating such strategies into existing surveillance systems could lead to earlier detection, better treatment planning, and enhanced long-term survivorship care.

Studies show that EC survivors face a significantly increased risk of developing secondary malignancies, which supports the need for expanded oncologic screening. In a population-based analysis, women diagnosed with EC had approximately a 71% higher risk of developing a second primary cancer compared to the general population, with more than a twofold increase in colorectal cancer risk and about a 40% increase in breast cancer risk [43]. Moreover, the incidence of colon cancer is markedly elevated in EC survivors, showing a standardized incidence ratio of approximately 2.5 [44,45,46]. These findings underscore the importance of implementing additional surveillance measures, such as routine colonoscopies and mammographies, to detect synchronous or metachronous malignancies in this population [44,46].

The presence of multiple primary tumors in a single patient strongly suggests an underlying hereditary predisposition [47,48]. Multiple primary neoplasms are frequently considered indicative of an inherited cancer syndrome [48], and genetic studies have confirmed this connection [49]. For example, in a recent multigene panel analysis, nearly one-third of patients with multiple primary tumors carried pathogenic germline variants, most commonly in BRCA1/2 and mismatch repair (MMR) genes associated with Lynch syndrome [50]. Consequently, current guidelines recommend systematic evaluation for hereditary syndromes in patients diagnosed with EC. Specifically, universal Lynch syndrome screening, using either targeted family history assessment or tumor-based molecular testing, is now recommended for all patients with EC, regardless of age or histologic subtype [51]. Additionally, BRCA1/2 mutation testing should be considered in patients whose personal or family history suggests a hereditary breast–ovarian cancer syndrome [52].

Furthermore, active management of metabolic comorbidities (obesity, diabetes mellitus, and hypertension) is necessary for prevention, prognosis, and treatment optimization. The rising prevalence of obesity has been a major contributor to the increasing incidence of EC in recent decades [53], with obesity estimated to account for up to 40% of all EC cases [54]. Intentional weight loss and sustained healthy body weight have been shown to significantly reduce EC risk among overweight or obese women [55]. Conversely, the presence of metabolic syndrome or diabetes at the time of EC diagnosis has been associated with poorer prognosis. A meta-analysis reported that EC patients with metabolic syndrome had significantly lower overall survival, with a ~57% higher mortality risk compared to those without [53]. Similarly, pre-existing diabetes was shown to double the risk of mortality and recurrence in EC patients compared to non-diabetic counterparts [56]. In long-term care, these comorbidities substantially contribute to overall mortality, with EC survivors being three times more likely to die from cardiovascular events than from gynecologic cancer recurrence [57,58]. Given the modifiable nature of these factors, multidisciplinary follow-up care should include interventions targeting weight control, blood pressure management, glucose regulation, and lifestyle counseling. Integrating such strategies into survivorship plans is vital, as many authors emphasize that aggressive management of metabolic conditions among EC survivors may significantly improve both quality of life and long-term survival [57,59].

This study has several important limitations that must be openly acknowledged. First, the relatively small sample size (*n* = 35) decreases the statistical power to detect subtle or moderate associations. This limitation increases the risk of false-negative findings. It also restricts the generalizability of our conclusions beyond this specific institutional cohort. Therefore, the results should be viewed as exploratory and mainly hypothesis-generating, needing confirmation in larger, prospective studies. Second, the retrospective and single-center design may have introduced selection and reporting biases, which subsequently limit the external validity and generalizability of our findings beyond the studied regional population. Although we evaluated associations with comorbidities and tumor characteristics, the lack of a control group consisting of patients with isolated endometrial carcinoma limits our ability to identify factors specific to cases with multiple primary malignancies.

Importantly, this study did not assess genetic predisposition, such as germline mutations associated with Lynch syndrome or BRCA1/2, which may play a central role in cases involving multiple primary malignancies. The absence of molecular or genetic profiling represents a significant limitation, especially given the current guidelines recommending universal screening for Lynch syndrome in EC patients. Future studies incorporating genetic data could better characterize etiological pathways and refine patient stratification.

Future research should aim to validate these findings in larger, multicenter cohorts including genetic profiling and long-term follow-up. The integration of germline and somatic mutation testing, alongside clinical and metabolic data, would allow for more accurate risk assessment and the identification of hereditary cancer syndromes. Additionally, the development of predictive models based on multivariate analysis should be tested prospectively to support personalized screening strategies. Expanding the research framework to include interventional studies focused on comorbidity management (e.g., weight reduction, glycemic control) may provide valuable insights into preventive strategies for reducing the burden of dual malignancies in EC survivors.

This study highlights the complexity of managing dual malignancies involving EC and reinforces the role of age and obesity as independent factors influencing tumor histology. While no clear comorbidity pattern was statistically validated, observed trends warrant further investigation in larger, controlled cohorts.

## 5. Conclusions

This study describes the clinical and pathological features of patients with dual primary malignancies involving endometrial carcinoma. Endometrioid histology was the predominant subtype and frequently co-occurred with breast and colorectal cancers. Although obesity, hypertension, and diabetes were common comorbidities, only age and obesity emerged as independent predictors of histology. At the same time, the interval between diagnoses showed a significant association with tumor characteristics, suggesting a potential pattern in tumor evolution. However, the small sample size (*n* = 35) and retrospective, single-center design limit statistical power and generalizability. These findings should therefore be interpreted with caution and regarded as hypothesis-generating. Larger, prospective studies are required to validate and expand these observations.

## Figures and Tables

**Table 1:** Demographic and clinical distribution of cases.

	Total *n* (%)	*p*-Value (chi-Square Test)	Adjusted *p*-Value (BH-FDR)
Total	35 (100%)		
Age (year)		0.9639	0.9639
Residential background		0.1753	0.31554
Urban	18 (51.44%)		
Rural	17 (48.56%)		
Uterine tumor type		0.0551	0.20571
Endometrioid	28 (80.00%)		
Mucinous intestinal-type	1 (2.85%)		
Clear cell	3 (8.57%)		
Serous	2 (5.71%)		
Mezonefric-like	1 (2.85%)		
Uterine Tumor Grade		0.3084	0.52084
Grade 1	6 (17.14%)		
Grade 2	21 (60.00%)		
Grade 3	8 (22.85%)		
FIGO stage		0.4051	0.52084
IA	5 (14.28%)		
IB	10 (28.57%)		
IC	4 (11.42%)		
IIA	3 (8.57%)		
IIB	2 (5.71%)		
IIIA	4 (11.42%)		
IIIB	3 (8.57%)		
IIIC	2 (5.71%)		
IVA	1 (2.85%)		
IVB	1 (2.85%)		
Associated tumors		0.0201	0.1752
Borderline seromucinous ovarian tumor	1 (2.85%)		
Endometrioid ovarian adenocarcinoma	3 (8.57%)		
Endometrioid borderline cystadenofibroma	1 (2.85%)		
Granulosa cell tumor	1 (2.85%)		
Papillary urothelial carcinoma	3 (8.57%)		
Serous ovarian adenocarcinoma	2 (5.71%)		
Hodgkin’s lymphoma	1 (2.85%)		
Invasive ductal breast carcinoma	10 (28.57%)		
Rectosigmoid adenocarcinoma	4 (11.42%)		
Squamous lung carcinoma	1 (2.85%)		
Malignant melanoma	2 (5.71%)		
Mesonephric-like ovarian adenocarcinoma	1 (2.85%)		
Small-cell neuroendocrine lung carcinoma	1 (2.85%)		
Typical carcinoid lung carcinoma	1 (2.85%)		
First malignancy		0.4014	0.52084
Uterine	8 (22.85%)		
Other organs	14 (40.00%)		
Synchronous tumors	12 (34.28%)		
Triple malignancy	1 (2.85%)		

**Table 2:** Age and interval-related associations between tumor pairs.

	Statistic	*p*-Value	Adjusted *p*-Value (BH-FDR)
Mann–Whitney U Test (Group 1 vs. Group 2)	U ^1^ = 51.0	0.7584	0.8532
Kruskal–Wallis Test (Interval vs. tumor characteristics)	H ^2^ = 6.91	0.0002	0.0036
Uterine Tumor Age Diagnostic	H ^2^ = 2.14	0.5435	0.6522
Associated Tumors Age Diagnostic	H ^2^ = 6.91	0.0747	0.20571

^1.^ U-statistic—Mann–Whitney U Test; ^2.^ H-statistic—Kruskal–Wallis Test.

**Table 3:** Demographic and clinical distribution of synchronous tumor cases.

	Total *n* (%)	*p*-Value	Adjusted *p*-Value (BH-FDR)
Total	13 (100%)		
Age (year)		0.9069	0.96024
Residential background		0.1540	0.308
Urban	3 (23.07%)		
Rural	10 (76.92%)		
Uterine tumor type		0.0292	0.1752
Endometrioid	11 (84.61%)		
Serous	1 (7.69%)		
Mezonefric-like	1 (7.69%)		
Uterine Tumor grade		0.3341	0.50115
Grade 1	2 (15.38%)		
Grade 2	8 (61.53%)		
Grade 3	3 (23.07%)		

**Table 4:** Results of the logistic regression model assessing the association between patient factors and the likelihood of endometrioid uterine tumor histology. Statistically significant predictors included age at diagnosis and obesity.

	OR ^3^	CI 95% ^4^ (2.5–97.5%)	Coefficient	*p*-Value
Age at diagnosis	0.88	0.79–0.98	−0.127	0.022
Obesity	0.11	0.01–0.83	−2.22	0.033
Type 2 Diabetes	0.9	0.12–6.68	−0.11	0.915

^3.^ OR—Odds Ratio; ^4.^ CI—Confidence Interval.

## Data Availability

The data supporting this study are available from the corresponding author upon request. However, they are not publicly accessible due to ethical considerations.
